# Targeting remaining pockets of malaria transmission in Kenya to hasten progress towards national elimination goals: an assessment of prevalence and risk factors in children from the Lake endemic region

**DOI:** 10.1186/s12936-019-2876-x

**Published:** 2019-07-12

**Authors:** Ismail Mahat Bashir, Nancy Nyakoe, Marianne van der Sande

**Affiliations:** 1 Department of Public Health, Institute of Tropical Medicine, Antwerp, Belgium; 2 Walter Reed Project, Kenya Medical Research Institute, Kisumu, Kenya; 3 Julius Global Health Center, Julius Center for Health Sciences and Primary Care, Utrecht University, Utrecht, The Netherlands

**Keywords:** Malaria, Parasitaemia, Prevalence, Risk factors, Endemicity, ITNs, Household survey, Kenya

## Abstract

**Background:**

With an overall decline of malaria incidence, elimination of malaria is gradually becoming the next target for many of countries affected by the disease. In Kenya the national malaria control strategy is aiming to reach pre-elimination for most parts of the country. However, considerable heterogeneity in prevalence of the disease within the country and especially the remaining high prevalent region of the Lake endemic region is likely to slow progress towards this target. To achieve a sustained control and an eventual elimination, a clear understanding of drivers of ongoing malaria transmission in remaining hotspots is needed.

**Methods:**

Data from the 2015 Malaria Indicator Survey (MIS) were analysed for prevalence of malaria parasitaemia in children (6 months to 14 years) of different countries within the highly endemic Lake region. Univariate and multivariate logistic regression analysis were preformed to explore associations between selected risk factors and being parasitaemic. A predictive model was built for the association between malaria and the risk factors with the aim of identifying heterogeneities of the disease at the lower administrative levels.

**Results:**

Overall, 604/2253 (27%, 95% CI 21.8–32.2) children were parasitaemic. The highest prevalence was observed in Busia County (37%) and lowest in Bungoma County (18%). Multivariate logistic regression analysis showed that the 10–14 years age group (OR = 3.0, 95% CI 2.3–4.1), households in the poorest socio-economic class (OR = 2.1, 95% CI 1.3–3.3), farming (OR = 1.4, 95% CI 1.2–2.5) and residence in Busia (OR = 4.6, 95% CI 2.1–8.2), Kakamega (OR = 2.6, 95% CI 1.3–5.4), and Migori counties (OR = 4.6 95% CI 2.1–10.3) were associated with higher risk of parasitaemia. Having slept under a long-lasting insecticide-treated bed net (LLIN) was associated with a lower risk (OR = 0.7, 95% CI 0.6–0.9). No association were found between malaria infection and the gender of the child, the household head, and the education status of the household head.

**Discussion and conclusion:**

Detailed analysis of malaria prevalence data in a hotspot area can identify new threats and avail opportunities for directing intervention. In the Lake endemic region of Kenya, interventions should be focused more on counties with the highest prevalence, and should target older children as well as children from the lower socio-economic strata. Precisely targeting interventions in remaining hotspots and high-risk populations will likely make impact and accelerate progress towards pre-elimination targets.

## Background

Kenya has witnessed a significant decrease in the prevalence of malaria over the past years. Parasitaemia declined from 11% in 2010 to 8% in 2015 [1]. This progress has led to a profound optimism to achieve pre-elimination targets, defined as period of re-orientation of malaria programme between the sustained control and elimination stages [2].

However, further progress in reducing or eliminating malaria will likely be challenging. This is related to a number of factors, including reduced funding for control activities as authorities lower priority on malaria following reducing prevalence and the emergence of resistance to insecticides used in indoor residual spraying (IRS), which has resulted in the discontinuation of indoor residual spraying (IRS) in the majority of Kenyan counties in the highest endemic region [3]. Furthermore, there is considerable heterogeneity in malaria transmission, with the prevalence of the disease still high in the expansive Western region along and around the Lake Victoria where prevalence stands at 27% as per 2015 Malaria Indicator Survey (MIS). Such persistent high prevalence hotspots hamper a national transition to a pre-elimination phase and will likely delay progress towards malaria elimination.

In order to implement targeted strategies to control malaria in this region, there is need to assess malaria risks at local levels to identify vulnerable populations and implement prevention strategies that are context-specific and tailored to address observed disparities in the prevalence of the disease. This requires understanding of the modifiable risk factors that increase the risk of disease in this region so that steps can be taken to mitigate them. Previous studies in settings similar to the Kenya has shown that factors such as age of child, socio-economic status of households, level of education of household head, housing conditions, land use, residence (rural *vs* urban), and sources of water for domestic use are risk factors associated with malaria parasitaemia [4–7]. At present, it remains unknown how these factors contribute to increased or decreased risks of malaria to individuals and households in different malaria endemicity regions in Kenya, especially in the declining phase of the disease. Such information, if available, would provide guidance to programme managers and policymakers in understanding the predictors of parasitaemia and in designing targeted control efforts to reduce malaria prevalence and accelerate progress towards pre-elimination, especially in the remaining endemic areas.

Using data collected during the MIS 2015, analysis of malaria parasitaemia prevalence by county (administrative areas consisting of multiple districts) within the Lake region of Kenya was performed. In addition, the associations between malaria parasitaemia in children and selected risk factors in this high endemic region was explored.

## Methods

### Study area

**Fig. 1:**
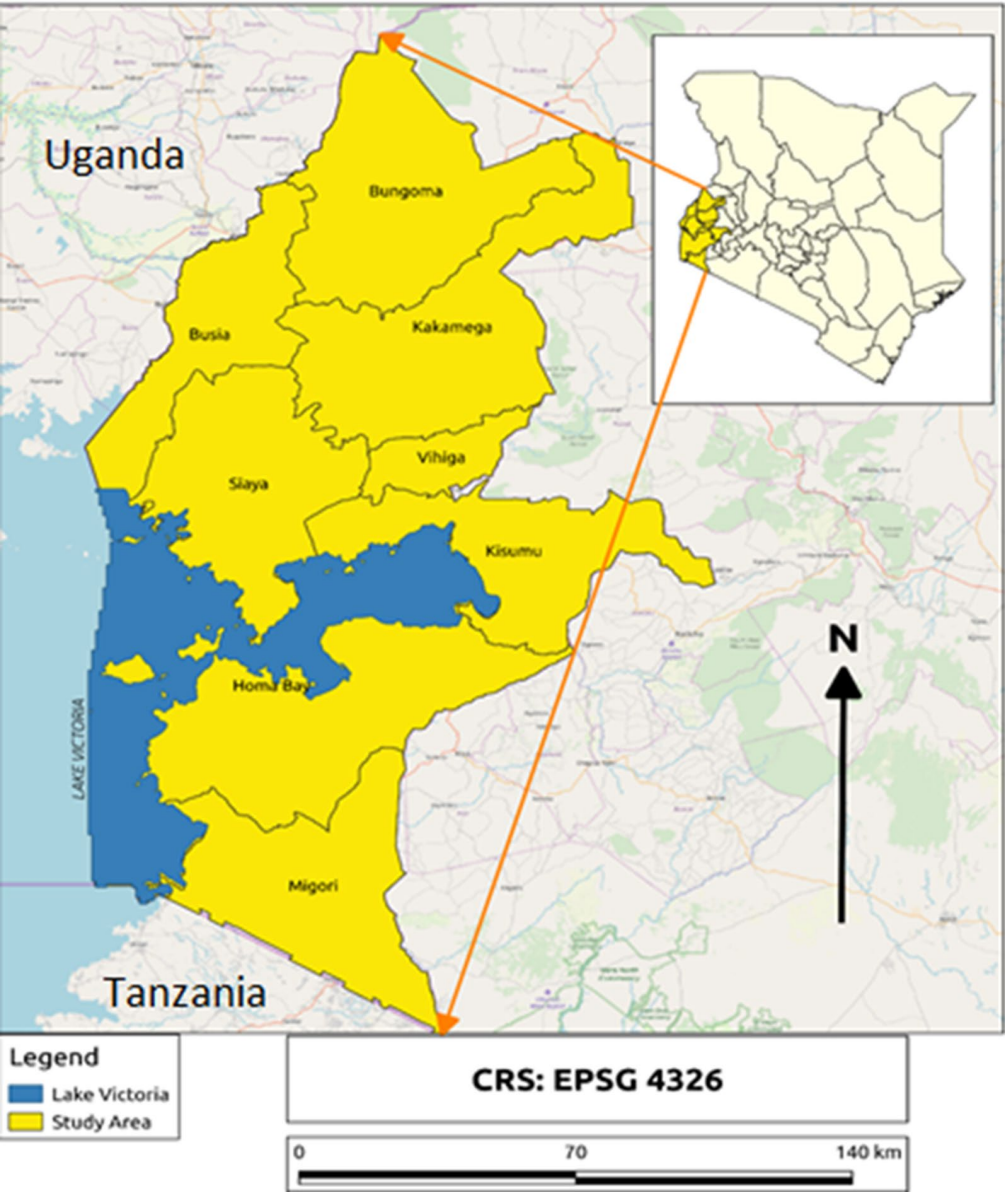
Study areas showing the counties with the Lake endemic region

The Lake endemic region is administratively made up of eight counties of Kisumu, Siaya, Migori, Homabay, Kakamega, Busia, Bungoma, and Vihiga (Fig. 1). With the exception of Kakamega, Vihiga and Bungoma, the counties in this region share a border with one of the largest freshwater lakes in Africa, Lake Victoria. Generally, the area has a tropical climate with average temperatures ranging from 19 to 27 °C, while altitude ranges from 1200 to 1700 m above sea level. With two rainfall patterns annually, mosquito vector population in this region are usually high and malaria transmission is intense due to suitable climate conditions. As a result, the region experiences stable malaria transmission throughout the year.

### Study design

Cross-sectional data collected during the 2015 Malaria Indicator Survey (MIS)—a household survey was analysed. Eligibility of households was determined through a representative probability sample designed to get independent estimates of the prevalence of malaria parasitaemia nationally, by endemicity zone and by urban/rural residence. Sampling involved a two-stage stratified cluster sampling. In the first stage, 246 clusters including 131 rural and 115 urban clusters were selected with equal probability from the country’s national sample survey and sampling programme [3]. The second stage involved systematically selecting 30 households from each cluster. In each household, all eligible women of childbearing age were invited for interview and only children of the women who were interviewed were included in the survey. Data were collected on characteristics of the household, including number of people in the house by age and gender, sanitation status, asset ownership, bed net ownership, and housing conditions. Thereafter, peripheral blood was collected from all children in the household aged 6 months to 14 years to test for malaria parasitaemia.

### Definitions

As a dependent variable in the logistic regression analysis, Parasitaemia was defined as the detection of any malaria parasite in a peripheral blood smear examined by microscopy. For most of the independent variables tested in our logistic regression, the definitions of the MIS 2015 without any alteration was used [3]. However, housing characteristics was defined as shown in Table 1.

**Table 1:** The definition of key variables on housing condition [8]

Variable	Definition
Roof material	Traditional = (thatch/grass/makuti (interwoven palm tree leaves), and dung/mud/sod) Modern = (iron sheets, asbestos, concrete, tiles)
Wall material	Traditional = (cane/palms/trunks, dung/mud/sod, bamboo with mud, re-used wood) Modern = (iron sheets, cement, stone with cement, bricks, blocks, covered adobe, and wood planks)
Floor material	Traditional = (Earth/sand, dung, wood planks, palm/bamboo). Modern = (Polished wood, Vinyl PVC, asphalt, ceramics, cement and carpet)

### Laboratory methods

MIS used rapid diagnostic tests (RDT) and microscopy to test for malaria in the selected children. For RDT, SD BIOLINE Malaria Ag P.f/Pan (Catalogue Number: 05FK60) was used. Briefly, blood was obtained by a finger prick, collected with a sterile loop applicator and placed in the RDT device. A buffer was added to the cassette so that the blood sample flows through the reaction area of the kit by capillary action. After 15 min, the results were read and recorded as either positive, negative or indeterminate. Indeterminate results were repeated until the test met the validity criteria.

Peripheral blood smears were collected and sent to a malaria diagnostic centre for staining and examination by an expert microscopist. At the malaria diagnostic centre, the thin smear was fixed with methanol and both the thick and thin blood smears were then stained in 20% Giemsa stained for 15 min. The smears were dried and examined microscopically with a high power objective. For quality assurance purposes, every slide was read by two trained microscopists at different times. A third reader was called to settle any discrepancy between the two readers.

As microscopy is considered the gold standard technique for the diagnosis of malaria, all analysis presented in this report are based on malaria microscopy results alone and not on RDT.

### Ethical considerations

This study involved the secondary analysis of data collected in 2015 and archived for further explorative analysis. No ethical approvals were necessary. Kenyatta National Hospital and University of Nairobi institutional review boards approved the primary protocol for MIS Kenya.

### Data analysis

Analysis was done with Stata version 12.0 software (STATA Corp, USA). Prevalence rates were assessed by counties and for children of different age groups. Univariate and multivariate logistic regressions were done to test for the association between malaria parasitaemia and selected risk factors, taking into account survey design effects. Microscopy confirmed malaria parasitaemia was used as dependent variable. Selected independent variables included the child’s age and gender, gender of the household head, socio-economic status of households defined by wealth index, house characteristics (modern *vs* traditional), sources of water for domestic use (pipe, wells, open sources), occupation of household head, and residence (urban *vs* rural).

To build a predictive model, all potential risk factors showing at least borderline significant statistical association with malaria (p < 0.1) were included in a multivariate logistic regression model. A stepwise backwards-elimination was used to identify final covariates in the model. Wald test was used to check for the effect of removing factors from the model. Factors maintained in the final model are presented as odds ratio plot with 95% confidence interval.

## Results

Nationally, MIS selected 7380 households but included 6667 (90.3%) in the final survey. A total of 10,721 equal to 93% of eligible children in the households participated [3]. In the Lake endemic region, 1290 households equivalent 20% of households that were sampled nationally and 2442 children equivalent to 22% of all the children selected nationally participated in the survey; the mean age was 89 months (7.4 years) and 50.2% were female; 89.3% of households reported ownership of at least one insecticide-treated net. Table 2 shows all the general characteristics of the children assessed in this report.

**Table 2:** General characteristics of children from Lake endemic region enrolled in MIS 2015

Characteristics	Number	%
Total number of children	2442	22.0
Age distribution		
Below 5 years	771	31.5
5–10 years	877	36.0
10–14 years	794	32.5
Gender		
Male	1228	49.8
Female	1214	50.2
Malaria		
Number tested by microscopy	2253	93
Children with malaria	604	27.3
Place of residence		
Rural	1661	78.6
Urban	781	21.4
Counties of origin		
Siaya	232	11.6
Kisumu	286	13.6
Migori	261	12.2
Homabay	321	13.4
Kakamega	330	14.6
Vihiga	233	11.0
Bungoma	272	10.9
Busia	292	12.1

**Fig. 2:**
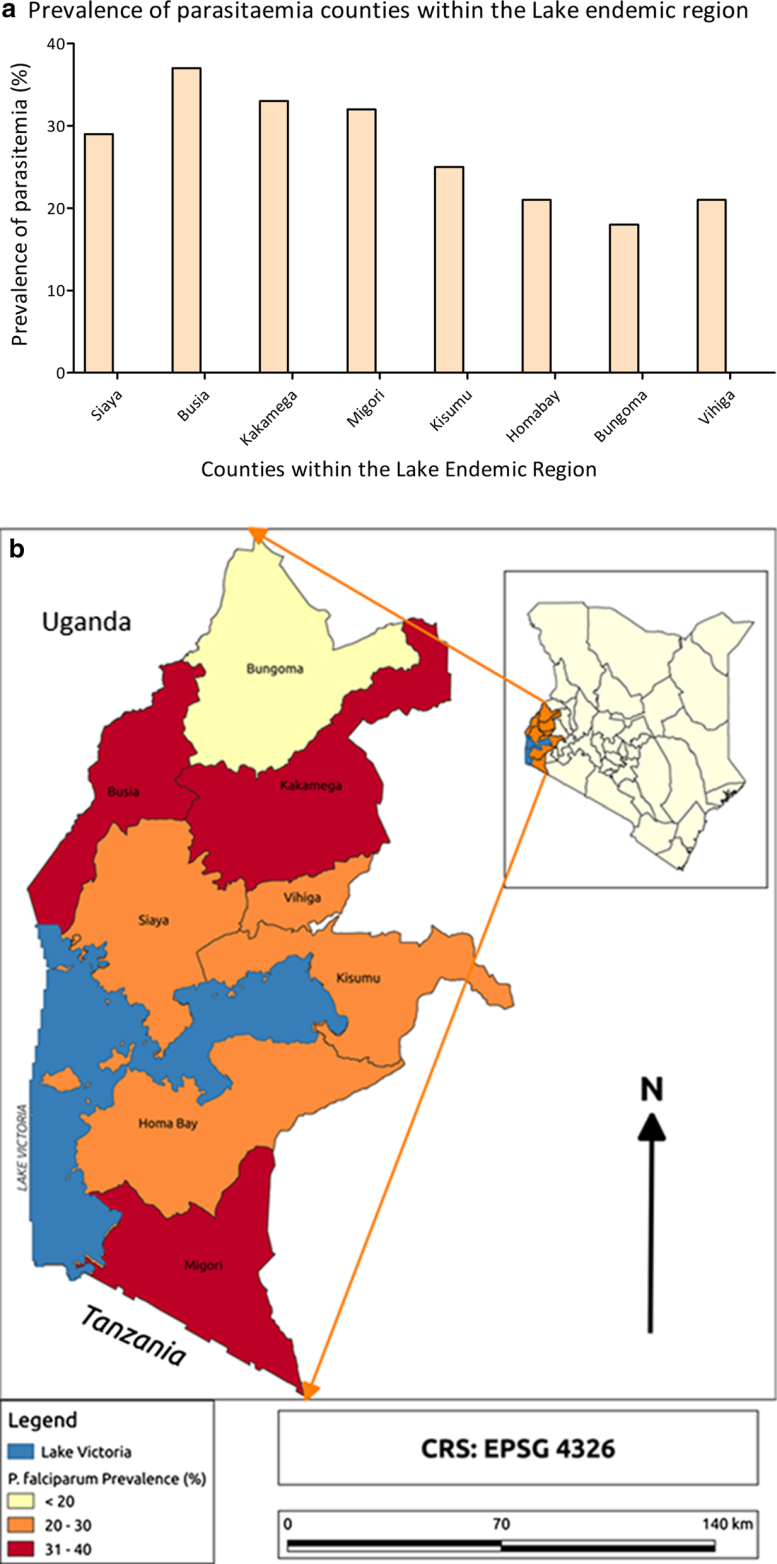
**a** Prevalence of parasitaemia in counties within the Lake endemic region (counties in random sequence). **b** Prevalence of parasitaemia in counties within the Lake endemic region

In general, of the 2253 children that were tested for malaria by microscopy, 604 (27.1%) tested positive compared to 8.0% positivity at national level. However, prevalence in the different counties was variable, ranging from highest 37% in Busia county to lowest 18% in Bungoma county (Fig. 2a). The counties of Busia and Migori, which border Lake Victoria, and Kakamega which is classified under the Highlands, had highest prevalence as shown in Fig. 2b.

**Fig. 3:**
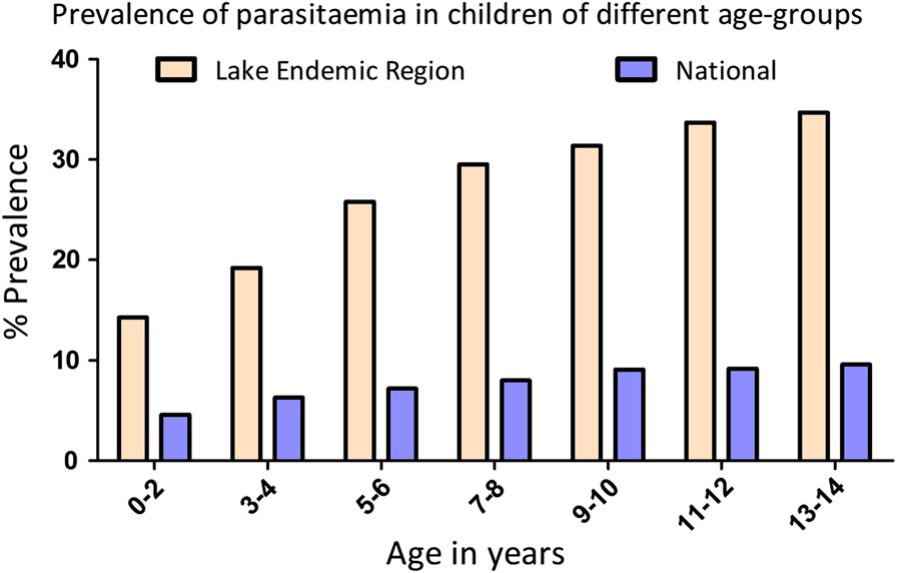
The prevalence of parasitaemia in different age categories in children from the Lake endemic region of Kenya compared to the national trend of malaria parasitaemia. Time points indicate 2-year age brackets

Regarding prevalence in children of different age groups, parasitaemia was lowest in the youngest age group, which had a mean parasitaemia of 14%. Gradually parasitaemia increased with age and reached a peak level of 35% at the age group of 13–14 years. The prevalence of parasitaemia in children from the Lake endemic region was relatively high compared to that of the national level at any given age group as shown in Fig. 3.

In univariate analysis, the main risk factors for parasitaemia were age of a child, socio-economic status, main materials for roof, floor and wall, practicing agriculture, residence (urban vs rural), and county of residence. In the multivariable analysis, only age, socio-economic status, agricultural farming and county of residence remained significantly associated with parasitaemia (Table 3).

**Table 3:** Univariate and multivariate analysis of the risk factors for malaria in children in the Lake endemic region in Kenya

Covariables	Multivariate analysis			
	Unadjusted		Adjusted	
	OR (95% CI)	*P*-value	OR (95% CI)	*P*-value
Age groups				
0–5 years	1 (Reference)		1	
5–10 years	2.03 (1.56–2.65)	*<0.001*	2.20 (1.69–2.87)	*< 0.001*
10–14 years	2.63 (1.93–3.58)	*<0.001*	3.04 (2.26–4.10)	*< 0.001*
Child’s gender				
Male	1			
Female	0.95 (0.80–1.12)	0.330		
Gender—household head				
Male	1		1	
Female	0.73 (0.53–1.01)	0.058	0.70 (0.51–0.96)	*0.030*
Education—household head				
No	1		1	
Yes	0.78 (0.58–1.06)	0.115	0.88 (0.69–1.13)	0.320
Wealth Index—SES				
Poorest	3.54 (1.93–6.50)	*<0.001*	2.06 (1.30–3.27)	*0.003*
Poorer	2.52 (1.49–4.26)	*0.001*	1.50 (0.93–2.41)	0.091
Middle	2.24 (1.45–3.46)	*0.001*	1.50 (0.99–2.29)	0.054
Rich	1		1	
Bed–net ownership				
Owns a bed net	0.67 (0.41–1.08)	0.101	0.74 (0.44–1.24)	0.251
Slept under LLIN	0.63 (0.47–0.85)	*0.004*	0.69 (0.55–0.87)	*0.002*
Floor material				
Modern	1		1	
Traditional	3.12 (2.58–3.78)	*<0.001*	1.62 (1.08–2.45)	*0.039*
Main wall material				
Modern	1		1	
Traditional	2.66 (2.19–3.23)	*<0.001*	1.64 (0.88–2.96)	0.115
Roofing material				
Modern	1		1	
Traditional	2.52 (1.90–3.34)	*<0.001*	1.54 (1.02–2.34)	*0.042*
Source of water				
Piped	1			
Wells/boreholes	1.32 (0.70–2.47)	0.378		
River/canal	1.33 (0.77–2.31)	0.302		
Does not practice agriculture	1		1	
Practices agriculture	2.34 (1.64–3.34)	*< 0.001*	*1.76 (1.23–2.52)*	*0.003*
Does not own a livestock	1		1	
Owns livestock	1.33 (0.98–1.81)	0.064	1.01 (0.74–1.39)	0.941
Residence				
Urban	1		1	
Rural	2.88 (1.40–5.92)	*0.005*	1.68 (0.96–2.95)	0.059
Counties				
Bungoma	1		1	
Siaya	2.45 (1.25–4.78)	*0.010*	3.40 (1.70–6.83)	*0.001*
Kisumu	1.64 (0.54–5.00)	0.378	2.58 (0.87–7.61)	0.085
Migori	2.54 (0.87–7.42)	0.086	4.64 (2.09–10.30)	*< 0.001*
Homabay	1.63 (0.84–3.16)	0.144	2.18 (1.13–4.24)	*0.022*
Kakamega	2.61 (1.18–5.79)	*0.019*	2.65 (1.29–5.43)	*0.009*
Vihiga	1.41 (0.45–3.25)	0.698	1.40 (0.48–4.07)	0.532
Busia	4.09 (1.91–8.75)	*0.001*	4.12 (2.07–8.19)	*< 0.001*

SES: Socio-economic status, *P* values less than 0.05 are highlighted in italics

Compared to children living in Bungoma County, which had the lowest observed prevalence, the adjusted odds of having malaria parasitaemia was significantly higher for children living in Migori (aOR = 4.64), Kakamega (aOR = 2.65) and Busia (aOR = 4.12).

**Fig. 4:**
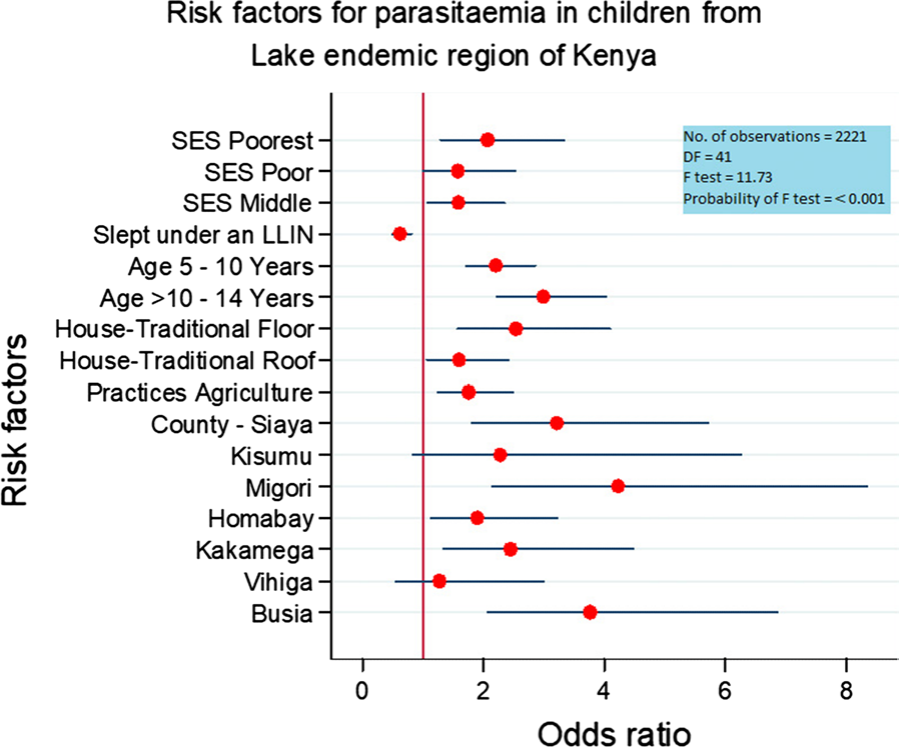
Plot of adjusted odds ratios and 95% confidence interval for risk factors in the final model in the Lake endemic malaria region of Kenya

To identify the main risk factors that drive malaria prevalence in the Lake endemic region, a predictive logistics regression model was built. Factors predicting increased risk for parasitemia included age group, wealth index, the county of residence, materials used in making roofs and floors of houses, and farming (see Fig. 4). Sleeping under a long-lasting insecticide-treated net (LLIN) predicted a decreased likelihood. There was no evidence of interaction between the factors in the model.

## Discussion

Using data from MIS 2015, prevalence and risk factors associated with parasitaemia in children from the Lake endemic region of Kenya was assessed. MIS is a highly standardized survey and is considered a gold standard surveillance tool to measure both the burden of malaria and the uptake of control strategies [3].

Analysis of MIS data has brought forth a number of important insights into the epidemiology of malaria in the Lake endemic region of Kenya. Firstly, despite the average malaria prevalence in this region standing at 27%, significant variations were evident in the levels of parasitaemia in children from different counties in the same region. Expectedly, the counties of Migori and Busia that border Lake Victoria had the highest prevalence relative to other counties in this region. This may be because environmental conditions around the Lake create conducive environments to sustain the mosquito vector and increase parasite transmission. A previous study from Mbita, a district on the shores of Lake Victoria has shown that important malaria vectors, such as *Anopheles funestus* and *Anopheles gambiae* breed on vegetation and in lagoons along the shores of the Lake [9]. The availability of water and breeding conditions throughout the year from this freshwater lake may provide a perennial source of transmission of malaria for these counties.

Surprisingly, Kakamega County, which is geographically considered a highland area but classified under the Lake endemic regions for operational convenience by National Malaria Control Programme (NMCP), had a malaria parasitaemia prevalence of 33%, the second highest in the region. This observation was unexpected as highlands areas are generally considered to have unsuitable environmental conditions for mosquito breeding and survival. Despite this, malaria in the highlands is increasingly becoming a health problem not only in Kenya but also in other sub-Saharan Africa countries such as Ethiopia, Rwanda and Burundi [10]. Such newly emerging risks need particular attention when moving towards pre-elimination phases, as they can jeopardize control efforts in the traditional key areas. It is therefore important to understand the factors fuelling transmission in this particular county so that preventive measures can be taken to interrupt ongoing transmission, avoid an outbreak and control the disease among the communities living within the region.

The differences in the levels of parasitaemia between the counties in the same endemic region require practical and policy considerations. In counties such as Migori, Kakamega and Busia, more than 30% of children tested were parasitaemic indicating a high level of exposure. Identifying these high-risk counties is important step towards implementation of targeted interventions. Considering the halted application of IRS in this region due to reported resistance to pyrethroids, the provision of universal access to bed nets alone, even though beneficial, may not be sufficient [3]. Strengthened environmental management and other integrated vector control strategies deployed simultaneously will likely have an impact in the control of vector population and reduce malaria transmission in these counties. In addition, it may be time to consider piloting the application of seasonal malaria chemoprophylaxis (SMC) for adults and older children with appropriate anti-malarials as a tool to fast track control towards pre-elimination, as feasibility and effectiveness of such programmes has been reported in Senegal [11].

The shift in the burden of the disease to older age groups reported here agrees with similar findings in Tanzania, Uganda and The Gambia [6, 8, 12]. It has been argued that as malaria declines, the dynamics of the disease changes, with older children and adults suffering most from the disease. Mathews et al. hypothesized that the numerous public health messages and specific interventions, such as LLINs, explicitly directed towards the under 5 years and pregnant women, which starts in the earliest term of the pregnancy and is continuous throughout the duration of pregnancy, may have contributed towards the protection of children under age five [6]. Other studies have proposed behavioural changes in the mosquito vector where the female *Anopheles* mosquito bites occur during early and late hours of the day as the reason for higher levels of parasitaemia in the older children who are likely to be playing outside [13].

The shift in the age burden to the older age groups requires strategies that address malaria in older children, as older children and adults may become the primary reservoirs of the parasite [14]. Given that older children are more likely schoolgoers, strategies are needed that target this group of children, including providing LLINs and ensuring consistent use through health educational programmes in schools. These efforts should happen without neglecting or reducing the current attention towards the under-fives. Importantly, the high prevalence of parasitaemia in children in areas with reported 80% of household ownership of a mosquito net and 75% consistent use according to MIS report 2015 [3] raises a fundamental question as to what extent malaria prevalence can be further reduced by LLIN use alone. As changes occur in mosquito-feeding behaviour, the shift in transmission time will require programmes that curtail transmission outdoors as a complement to those that prevent malaria indoors so that maximum reduction in transmission can be achieved. A potential strategy that could act as an adjunct to universal ITN use is the application of intermittent preventive therapy for older children (IPTc) with an effective long-acting anti-malarial drug to purposely curtail the effects of chronic parasitaemia in this important group. While the effectiveness of IPTc using combination of different anti-malarial drugs in younger children in this region has been reported by Odhiambo et al. the challenge may lie in timing its application as their effectiveness has been shown to wane [15].

Wealth index and the nature of materials used for the roof, the wall and the floor of houses, which are both considered proxy measures of socio-economic status of households, have been shown to have an association with being parasitaemic. The richer households enjoy a purchasing power which allows them to not only dwell in better conditioned houses but also enables them to afford a LLIN so that exposure of their household members to mosquitoes is lowered and risk of getting malaria reduced. These findings collaborate with the widely held view that malaria is a disease of poverty at the household levels. Similar findings relating to the associations between household socio-economic status and malaria have been reported in other studies in sub-Saharan Africa [7, 8, 16, 17]. Contrary to this result, Pullan et al. and Snow et al. who did similar studies in Uganda and Kenya, respectively, did not find any association between socio-economic status and malaria [18, 19]. However, the difference between this report and those with similar findings compared to these two studies with different results are that the latter was conducted in single communities living in rural settings. As a result, asset index data collected in such a small and rural setting may not be comparable to MIS that encompasses both urban and rural populations as well as a mix of different communities.

Children whose households reported that they slept under LLIN had 30% lower likelihood of having parasitaemia after adjustment for age, socio-economic status and other factors. Like other studies of similar findings, this result adds to the current evidence of the efficacy of LLINs which are the mainstay of malaria prevention. The modest protective effect of LLINs reported here against parasitaemia is similar to many other findings in different studies [5, 12, 18, 20].

Malaria parasitaemia was also associated with households that practised agricultural farming. People living in the Lake endemic region of Kenya generally practice large-scale farming of cane and rice and engage in small-scale farming of corn and other vegetations, which all create the right breeding environment for the mosquito vector. This increased occupational risk for farmers is in agreement with many other findings reported previously [8, 21, 22]. To mitigate risk related to farming, which remains one of the major economic activities for people of the Lake endemic region, it is essential that Kenya’s NMCP programme integrates environmental management programmes, including community managed larval control programmes with routine agricultural practices. Lessons on this can be drawn from the implementation of community-based larviciding activities implemented in Dar es Salaam, Tanzania, which has reported the successful outcome in the reduction of malaria cases in that region [23].

Even though the design of MIS is generally suitable for the analysis carried out in this study, potential limitations within the data need to be noted. Firstly, like all studies based on a cross-sectional survey, the associations observed may be unsuitable to draw causal relationship as they may be confounded by unmeasured factors [20]. Secondly, socio-economic status described in this report uses wealth index that was designed using principal component analysis (PCA) of assets in the households. It has been stated that expenditure data may be a better measure of how rich or poor households are in a survey, compared to wealth index measurement using PCA [19]. Lastly, the result of the malaria microscopy test was used to measure parasitaemia, i.e., dependent variable, even though it is known that microscopy is a less sensitive method compared to molecular techniques [24]. For MIS Kenya, examination of slides by two research microscopist with the quality assurance processes that included the confirmation of slide status by an expert microscopist improved the sensitivity of the technique.

## Conclusion

Despite the positive progress made by NMCP in the control of malaria in Kenya in the last decades, there are still some regions of the country where prevalence of parasitaemia is very high and heterogenous. It is therefore imperative to ensure that the current malaria control tools are deployed to get a maximum impact by targeting the areas and individuals who are at greater risk of the disease. Designing control strategies aimed at protecting school-age children and adults and implementing targeted interventions in counties with higher burden may be a useful strategy to make an impact on the levels of transmission of the parasite, to try to achieve nationwide pre-elimination.

## Data Availability

Data was provided by Demographic and Health Survey—USAID project.

## Abbreviations

IPTc: intermittent preventive therapy—children
IRS: indoor residual spray
ITN: Insecticide Treated Nets
IVM: integrated vector management
LLIN: Long-Lasting Insecticide Treated Nets
MIS: Malaria Indicator Survey
NMCPP: National Malaria Control and Prevention Programme
OR: odds ratio
PCA: principal component analysis
RDT: rapid diagnostic tests
SES: socio-economic status
SSA: sub-Saharan Africa
WHO: World Health Organization
